# The association between green space and depressive symptoms in pregnant women: moderating roles of socioeconomic status and physical activity

**DOI:** 10.1136/jech-2015-205954

**Published:** 2015-11-11

**Authors:** R R C McEachan, S L Prady, G Smith, L Fairley, B Cabieses, C Gidlow, J Wright, P Dadvand, D van Gent, M J Nieuwenhuijsen

**Affiliations:** 1 Bradford Institute for Health Research, Bradford Teaching Hospitals NHS Foundation Trust, Bradford, UK; 2 Department of Health Sciences, University of York, York, UK; 3 Institute for Environment and Sustainability Research, Staffordshire University, Stoke-on-Trent, UK; 4 Division of Epidemiology and Biostatistics, University of Leeds, Leeds, UK; 5 Faculty of Medicine- Clínica Alemana de Santiago, Universidad del Desarrollo, Santiago, Chile; 6 Centre for Sport, Health and Exercise Research, Staffordshire University, Stoke-on-Trent, UK; 7 Centre for Research in Environmental Epidemiology (CREAL), Barcelona, Spain

**Keywords:** DEPRESSION, DEPRIVATION, Environmental epidemiology, ETHNICITY, PREGNANCY

## Abstract

**Background:**

The current study explored the association between green space and depression in a deprived, multiethnic sample of pregnant women, and examined moderating and mediating variables.

**Method:**

7547 women recruited to the ‘Born in Bradford’ cohort completed a questionnaire during pregnancy. A binary measure of depressive symptoms was calculated using a validated survey. Two green space measures were used: quintiles of residential greenness calculated using the normalised difference vegetation index for three neighbourhood sizes (100, 300 and 500 m buffer zones around participant addresses); access to major green spaces estimated as straight line distance between participant address and nearest green space (>0.5 hectares). Logistic regression analyses examined relationships between green space and depressive symptoms, controlling for ethnicity, demographics, socioeconomic status (SES) and health behaviours. Multiplicative interactions explored variations by ethnic group, SES or activity levels. Mediation analysis assessed indirect effects via physical activity.

**Results:**

Pregnant women in the greener quintiles were 18–23% less likely to report depressive symptoms than those in the least green quintile (for within 100 m of green space buffer zone). The green space-depressive symptoms association was significant for women with lower education or who were active. Physical activity partially mediated the association of green space, but explained only a small portion of the direct effect.

**Conclusions:**

Higher residential greenness was associated with a reduced likelihood of depressive symptoms. Associations may be stronger for more disadvantaged groups and for those who are already physically active. Improving green space is a promising intervention to reduce risk of depression in disadvantaged groups.

## Introduction

Depression is a leading cause of disability globally.^1^ Approximately 12% of women experience depression during pregnancy,^2^ which can increase risk of adverse pregnancy outcomes, including preterm birth, low birth weight, being small for gestational age and reduced initiation of breast feeding.^3–5^ Depression in pregnancy is also the largest risk factor for postnatal depression, which affects the subsequent health and well-being of children.^6^ ^7^

Environmental characteristics are now recognised as possible determinants of depression.^8^ Lack of evidence for a relationship between green space and mental health in adults has been attributed, in part, to inconsistent and limited tools to measure green space.^9^ The ‘Normalised Difference Vegetation Index’ (NDVI), an assessment of the proportion of the photosynthetically active land cover from satellite images, has been recommended as a way to standardise green space assessment, allowing cross-study comparisons.

Among the growing green space-health literature, several studies have explored associations between green space and birth weight^10–13^ or neonatal mortality.^14^ To the best of our knowledge, there are no studies examining the relationship between green space and depression in pregnant women. A number of mechanisms are proposed to mediate relationships between green space and health. These include improved air quality, psychological restoration and stress reduction, increased opportunities for social contacts and physical activity.^15^ Recently, research has called into question the role of physical activity as a mediator of this relationship.^16–18^ However, a recent study found a moderating role of physical activity where the beneficial effects of green space were stronger for more active individuals.^19^ Whether this reflects increased exposure to green space (eg, visiting green spaces more regularly) or by enhanced physiological benefits of physical activity^20^ is, however, unclear.

The beneficial effects of green space may be moderated by socioeconomic status (SES), with greater benefits seen in lower socioeconomic groups, although the literature reports conflicting findings.^10^ ^11^ ^19^ ^21^ ^22^ Emerging findings also suggest that ethnicity can moderate relationships between green space and health.^11^ The aim of this paper was to explore whether residential ‘greenness’ and access to green space were associated with depressive symptoms among pregnant women. Secondary aims were to explore whether associations varied by ethnicity and SES. Finally, we aimed to explore whether physical activity mediated the relationship between green space and depressive symptoms.

## Method

### Study design

This study used the ‘Born in Bradford’ (BiB) birth cohort, a longitudinal cohort of 12 453 mothers (and 13 818 children) who were recruited at 28 weeks gestation, from 2007 to 2011. A full description of the cohort and setting has been reported elsewhere.^23^ Ethical approval was obtained from Bradford Research Ethics Committee (reference 07/H1302/112).

### Participants

Women with singleton pregnancies (N=7547) who completed the baseline questionnaire at recruitment from September 2007 to December 2010, and had complete data for all variables in the analyses.

### Variables

#### Primary outcome

Depressive symptoms were assessed using the ‘severe depression’ subscale of the General Health Questionnaire (GHQ-28).^24^ Our previous research has found differences in the way in which GHQ items are interpreted across cultural groups, which can cause difficulties for cross-cultural comparisons.^25^ Thus, we used a subset of four questions which we have found consistently relate to the same latent construct (depression) across different cultural groups.^25^ These were used in the current study as a proxy indicator of depression. Questions used the stem ‘over the past few weeks…have you’ and asked whether respondents: (1) felt life was entirely hopeless; (2) felt that life was not worth living; (3) found at times they could not do anything because their nerves were too bad (all scored 0: not at all to 3: much worse than usual); (4) thought of the possibility that they might make away with themselves (scored 0: definitely not to 3: definitely have). The binary depressive symptoms variable was defined as ‘not reporting any symptoms’ (where scores to all questions were 0) versus ‘reporting depressive symptoms’ (where participants scored 1 or above on *any* item).

#### Exposure to green space

Residential surrounding greenness was calculated using the NDVI within three straight line buffer distances of 100, 300 and 500m around participants' geocoded home addresses. The NDVI map was created using two images (10/06/2006 and 28/09/2011, selected as these had least cloud cover) obtained from the Earth Observing System Data and Information System website (https://earthdata.nasa.gov/). The NDVI ranges between −1 and +1, with higher values indicating more green vegetation. As this is an index with an arbitrary range it is difficult to interpret the importance of absolute values. Therefore, relative differences were explored using quintiles, where 1=least green quintile (mean NDVI=0.28, SD=0.36) and 5=greenest quintile (mean NDVI=0.60, SD=0.05).

Access to a major green space larger than 0.5 hectares (5000 m^2^) was measured using straight line distances between home addresses to the boundary of the nearest major green space, identified from Urban Atlas (http://www.eea.europa.eu/data-and-maps/data/urban-atlas). Participants were dichotomised as either having versus not having access to a major green space within 300 m.^26^

#### Covariables

##### Ethnicity

Ethnicity was self-reported at baseline and categorised into four groups considering the language of questionnaire administration: (1) White British origin, English language; (2) South Asian origin, English language; (3) South Asian origin, Urdu/Mirpuri language, (4) Other, English language. This was in accordance with the findings of Prady *et al,*^25^ who found measurement differences in reports of well-being dependent on ethnolanguage group.

##### SES indicators

SES was measured at individual and area level following Prady *et al*.^27^ Individual indicators included maternal education (highest educational qualification), and a subjective measure of poverty (‘How well would you say you or you and your husband/partner are managing financially these days’).^28^ At an area level, National Index of Multiple Deprivation^29^ quintiles were mapped to lower super output areas (1=most deprived to 5=least deprived) based on postcode of residence.

##### Physical activity

Physical activity was assessed using the general practice physical activity questionnaire.^30^ Respondents were coded as inactive, moderately inactive, moderately active or active.

#### Others

Additional control variables included: age at recruitment; parity; marital and cohabitation status; tertiles of household size (calculated within each ethnolanguage group); smoking; and alcohol use during pregnancy.^27^

### Statistical methods

Unadjusted logistic regression models were first computed using green space quintiles (reference group: 1 least green) as a predictor, and binary depressive symptoms as the outcome. Control variables were entered sequentially to adjust, first, for ethnolanguage grouping (model 2), then demographics (model 3: age, parity, marital and cohabitation status), SES (model 4: education, financial struggles, household size, index of multiple deprivation, IMD) and health behaviours (model 5: smoking, alcohol use and physical activity). Analyses were conducted for all three buffer sizes (100, 300 and 500 m), and then repeated using the binary ‘access to green space’ variable as a predictor. All variables were categorical with the exception of IMD quintile, which was entered as an ordinal variable.

To explore moderating roles of ethnicity, SES and physical activity, we entered separate interaction terms to an unadjusted model, one at a time for each green space buffer zone. For parsimony, we collapsed the ethnolanguage variable into three categories (White British, South Asian: both English and Urdu/Mirpuri administered; and Other), and the physical activity variable into inactive (inactive/moderately inactive) and active (moderately active/active). For SES we entered education, financial struggles and IMD quintile. We statistically tested interactions using the likelihood ratio test; where these were statistically significant, we stratified the full adjusted model to explore patterns.

Binary mediation within Stata^31^ was used to explore the mediating role of a binary measure of physical activity. To increase power, we included the continuous measure of NDVI as the independent variable. Bootstrapping was used to create SEs and 95% CIs. We repeated the analyses for all buffer sizes.

## Results

### Participants

The 7547 participants who had complete data represented 78.4% of the BiB cohort; there were no differences in key demographics between the groups (see online supplementary file table S1, and figure S1 for comparison table and flow diagram). A third of the sample (n=2530) reported at least one depressive symptom (table 1). Mean NDVI was highest for White British participants and lowest for South Asian Urdu/Mirpuri participants across all buffers (mean 0.50–0.54 vs 0.39–0.44, respectively). Only 9% of South Asian Urdu/Mirpuri participants lived in the greenest quintile of within 100 m of green buffer zone, compared with 30% of White British participants. Overall, 81% lived within 300 m of a large green space (>0.5 hectares); again, this was highest for White British participants (90%) and lowest for South Asian Urdu/Mirpuri participants (70%).

**Table 1:** Characteristics of study participants split by Ethnolanguage group

	Total N=7547	White British–English N=3079	South Asian–English N=2145	South Asian–Urdu or Mirpuri N=1262	Other ethnic groups–English N=1061
Depression symptoms					
No	5017 (66.5)	2270 (73.7)	1136 (53.0)	1026 (81.3)	585 (55.0)
Yes	2530 (33.5)	809 (26.3)	1009 (47.0)	236 (18.7)	476 (44.9)
NDVI (m)					
100	0.45 (0.11)	0.50 (0.09)	0.41 (0.11)	0.39 (0.10)	0.45 (0.11)
300	0.48 (0.10)	0.53 (0.09)	0.44 (0.10)	0.42 (0.09)	0.47 (0.10)
500	0.49 (0.10)	0.54 (0.09)	0.45 (0.09)	0.44 (0.08)	0.48 (0.10)
Least green quintile (100 m buffer, mean NDVI 0.28)	1457 (19.3)	151 (4.9)	651 (30.4)	471 (37.3)	184 (17.3)
Most green quintile (100 m buffer, mean NDVI 0.60)	1541 (20.4)	978 (30.1)	290 (13.5)	108 (8.6)	215 (20.3)
Access to green space within 300 m?					
No	1457 (19.2)	350 (11.4)	550 (25.6)	385 (30.5)	166 (15.7)
Yes	6092 (80.7)	2727 (88.6)	1594 (74.3)	877 (69.5)	894 (84.3)
Missing	4 (0.1)	2 (0.1)	1 (0.1)	0	1 (0.1)
*Demographic variables*					
Maternal age (years)					
<21	862 (11.4)	549 (17.8)	154 (7.18)	55 (4.4)	104 (9.8)
21–34	5782 (76.6)	2154 (70.0)	1766 (82.3)	1046 (82.9)	816 (76.9)
≥35	903 (12.0)	376 (12.2)	225 (10.5)	161 (12.8)	141 (13.3)
Parity					
0	3016 (40.0)	1432 (46.5)	728 (33.9)	355 (28.1)	501 (47.2)
1–2	3411 (45.2)	1362 (44.2)	1001 (46.7)	589 (46.7)	459 (43.3)
≥3	1120 (14.8)	285 (9.3)	416 (19.4)	318 (25.2)	101 (9.5)
Married and living with partner	4904 (65.0)	983 (31.9)	1950 (90.9)	1213 (96.1)	758 (71.4)
Not married and living with partner	1376 (18.2)	1227 (39.9)	12 (0.6)	2 (0.2)	135 (12.7)
Not living with partner	1267 (16.8)	869 (28.2)	183 (8.5)	47 (3.7)	168 (15.8)
*Socioeconomic status*					
Maternal Education					
Low	4436 (58.8)	1940 (63.0)	1098 (51.2)	922 (73.0)	476 (44.9)
High	3111 (41.2)	1139 (37.0)	1047 (48.9)	340 (26.9)	585 (55.1)
Subjective poverty					
Struggling financially	2370 (31.4)	1021 (33.2)	590 (27.5)	454 (36.0)	305 (28.7)
Not struggling financially	5177 (68.6)	2058 (66.8)	1555 (72.5)	808 (64.0)	756 (71.3)
Household size: tertiles of household size within four group ethnic categorisations					
Bottom tertile	1414 (18.7)	105 (3.4)	658 (30.7)	298 (23.6)	353 (33.3)
Middle tertile	2532 (33.6)	1057 (34.3)	764 (35.6)	397 (31.5)	314 (29.6)
Top tertile	3601 (47.7)	1917 (62.3)	723 (33.7)	567 (44.9)	394 (37.1)
Index of multiple deprivation					
Bottom national quintile	5373 (65.9)	1573 (51.1)	1643 (76.6)	1066 (84.5)	691 (65.1)
*Health behaviours*					
Alcohol use: drank alcohol during pregnancy or 3 months before					
Yes	2380 (31.5)	2068 (67.2)	9 (0.4)	1 (0.1)	302 (28.5)
No	5167 (68.5)	1011 (32.8)	2136 (99.6)	1261 (99.9)	759 (71.5)
Smoking during pregnancy					
Yes	1348 (17.9)	1101 (35.8)	117 (5.5)	10 (0.8)	120 (11.3)
No	6199 (82.1)	1978 (64.2)	2028 (94.5)	1252 (99.2)	941 (88.7)
Physical activity					
Inactive	4425 (58.6)	1353 (43.9)	1381 (64.4)	1201 (95.2)	490 (46.2)
Moderately inactive	1641 (21.7)	810 (26.3)	485 (22.6)	41 (3.3)	28.8 (305)
Moderately active	1153 (15.3)	703 (22.8)	222 (10.3)	18 (1.4)	210 (19.8)
Active	328 (4.4)	213 (6.9)	56 (2.7)	2 (0.2)	56 (5.3)

N and % reported in parentheses for categorical variables, mean and SD in parentheses reported for continuous NDVI variables.

NDVI, Normalised Difference Vegetation Index.

In the least green quintile, 39% of women reported depressive symptoms, compared with 31% in the greenest quintile. For those reporting depressive symptoms, mean distance to the nearest large green space was 189 m (SD 134 m), compared with 173 m (SD 130 m) for those not reporting depressive symptoms (see online supplementary table S2).

### Is green space associated with reporting of depressive symptoms during pregnancy?

Table 2 reports unadjusted and adjusted models of associations between green space quintiles and depression in pregnant women. Compared with those in the least green areas, those in greener quintiles were significantly less likely to report depressive symptoms. Associations were strongest within a 100 m green buffer zone, and protective effects persisted after adjustment for all variables. Within the 100 m green buffer zone, after adjustment, those in the greener quintiles (quintiles 3, 4 and 5) were 18–23% less likely to report depressive symptoms than those in the least green quintile areas.

**Table 2:** Association between NDVI and reporting of depressive symptoms

	NDVI 100 m	NDVI 300 m	NDVI 500 m
Model 1: Unadjusted†			
Quintile 2	0.87 (0.75 to 1.01)	0.87 (0.75 to 1.02)	0.88 (0.76 to 1.03)
Quintile 3	0.71 (0.61 to 0.82)***	0.68 (0.59 to 0.79)***	0.68 (0.59 to 0.80)***
Quintile 4	0.68 (0.59 to 0.79)***	0.71 (0.61 to 0.82)***	0.73 (0.63 to 0.85)***
Quintile 5 (greenest)	0.71 (0.61 to 0.82)***	0.64 (0.55 to 0.75)***	0.59 (0.51 to 0.69)***
Model 2: Adjusted for ethnicity‡			
Quintile 2	0.89 (0.76 to 1.04)	0.90 (0.77 to 1.05)	0.93 (0.79 to 1.08)
Quintile 3	0.73 (0.62 to 0.86)***	0.74 (0.63 to 0.87)***	0.77 (0.66 to 0.91)**
Quintile 4	0.74 (0.63 to 0.87)***	0.77 (0.65 to 0.90)**	0.78 (0.67 to 0.92)**
Quintile 5 (greenest)	0.76 (0.65 to 0.90)**	0.71 (0.60 to 0.84)***	0.66 (0.56 to 0.78)***
Model 3: As model 2+demographics§			
Quintile 2	0.87 (0.75 to 1.02)	0.89 (0.76 to 1.04)	0.92 (0.79 to 1.08)
Quintile 3	0.73 (0.62 to 0.86)***	0.75 (0.64 to 0.88)**	0.78 (0.66 to 0.92)**
Quintile 4	0.73 (0.62 to 0.86)***	0.75 (0.64 to 0.89)**	0.79 (0.67 to 0.92)**
Quintile 5 (greenest)	0.76 (0.64 to 0.89)***	0.74 (0.62 to 0.87)***	0.71 (0.60 to 0.85)***
Model 4: As model 3+socioeconomic indicators¶			
Quintile 2	0.89 (0.76 to 1.05)	0.91 (0.78 to 1.07)	0.95 (0.80 to 1.11)
Quintile 3	0.78 (0.66 to 0.92)**	0.81 (0.68 to 0.96)*	0.84 (0.71 to 0.999)*
Quintile 4	0.78 (0.65 to 0.93)**	0.82 (0.69 to 0.98)*	0.85 (0.72 to 1.01)
Quintile 5 (greenest)	0.82 (0.69 to 0.98)*	0.84 (0.70 to 1.01)	0.81 (0.68 to 0.98)*
Model 5: As model 4+health behaviours††			
Quintile 2	0.89 (0.76 to 1.05)	0.90 (0.77 to 1.06)	0.95 (0.81 to 1.11)
Quintile 3	0.77 (0.65 to 0.91)**	0.80 (0.67 to 0.95)*	0.84 (0.71 to 0.996)*
Quintile 4	0.77 (0.65 to 0.92)**	0.81 (0.68 to 0.97)*	0.85 (0.71 to 1.00)
Quintile 5 (greenest)	0.82 (0.69 to 0.98)*	0.84 (0.70 to 1.00)	0.81 (0.67 to 0.98)*

Odds radio and 95% CI reported; Quintile 1 (least green quintile) reference category.

Quintile 1 is the least green quintile, and serves as the reference category; quintile 5 is the most green quintile.

*p<0.05, **p<0.01, ***p<0.001.

†n=7547.

‡Adjusted by ethnolanguage group.

§Model 2+age, parity, marital and cohabitation status.

¶Model 3+education, financial struggles, household size, IMD index of multiple deprivation.

††Model 4+smoking, alcohol use and physical activity.

NDVI, Normalised Difference Vegetation Index.

A similar pattern occurred for access to green space. After adjustment, those who were within 300 m of a major green space were 13% less likely to report depressive symptoms (model 5, table 3).

**Table 3:** Association between access to green space and risk of depression

	Model 1†	Model 2‡	Model 3§	Model 4¶	Model 5††
Access to green space Yes	0.79 (0.71 to 090)***	0.81 (0.72 to 0.92)**	0.82 (0.73 to 0.94)**	0.88 (0.77 to 0.999)*	0.87 (0.77 to 0.995)*

Odds radio and 95% CI reported; No access to green space within 300 m as reference category.

Three decimals places are used for clarity when reporting upper 95% CI estimates for models 4 and 5.

*p<0.05, **p<0.01, ***p<0.001.

†n=7543.

‡Adjusted by ethnolanguage group, n=7486.

§Model 2+age, parity, marital and cohabitation status, n=7486.

¶Model 3+education, household size, IMD index of multiple deprivation, n=7486.

††Model 4+smoking, alcohol use and physical activity, n=7486.

### Do associations vary by ethnicity or SES?

We then explored whether associations between green space quintile and depression varied according to ethnicity or SES. To avoid problems of low numbers in the less deprived IMD quintiles, we aggregated quintiles 3–5 to give three groups (1: extremely deprived – lowest national quintile; 2: very deprived – second lowest national quintile; 3+: least deprived – comprising national quintiles 3–5). We tested the interaction term within the 100m buffer zone only as there were very few individuals in the 3+ category who lived within the lowest quintile of green space. There were no statistically significant interactions between financial struggles, IMD, ethnolanguage grouping and residential greenness using the continuous NDVI measure (results not shown).

A significant interaction was apparent for education status within 300 m of the green space buffer zone (likelihood ratio test: p=0.04). Unadjusted and fully adjusted stratified models are reported for within the 300 m green space buffer zone (table 4). Data for within 100 m and 500 m of the green space buffer zones are not shown, although the pattern of results was similar. In unadjusted models, the protective effect of living in a greener area appeared similar for low and high education groups. However, after controlling for demographics, SES and health behaviours, a statistically significant positive relationship between green space quintile and depression was apparent only in the low education group. For these individuals, being in the greenest quintile was associated with a 26% reduction (OR 0.74, 95% CI 0.59 to 0.94) in reporting of depressive symptoms compared with the least green quintile. There were no statistically significant differences for those in the high education group. No statistically significant interactions with ethnolanguage group or SES indicators were observed for access to green space (results not shown).

**Table 4:** Stratified models for relationship between NDVI and depression (300 m buffer zone) stratified by maternal education (top half of table) and physical activity (bottom half of table)

Education status	Low†	High‡
Model 1: Unadjusted		
Quintile 2	0.76 (0.63–0.92)**	1.12 (0.87–1.44)
Quintile 3	0.70 (0.57–0.984)***	0.68 (0.53–0.88)**
Quintile 4	0.70 (0.58–0.84)***	0.75 (0.59–0.96)*
Quintile 5 (greenest)	0.69 (0.57–0.84)***	0.64 (0.50–0.81)***
Model 5: Adjusted for ethnicity, demographics, SES and health behaviours		
Quintile 2	0.77 (0.63–0.94)*	1.18 (0.90–1.54)
Quintile 3	0.72 (0.58–0.90)**	0.92 (0.70–1.21)
Quintile 4	0.71 (0.57–0.88)***	1.00 (0.76–1.33)
Quintile 5 (greenest)	0.74 (0.59–0.94)*	1.00 (0.75–1.34)

Physical activity	Inactive§	Active¶
Model 1: Unadjusted		
Quintile 2	0.92 (0.79–1.08)	0.62 (0.41–0.93)
Quintile 3	0.71 (0.60–0.84)***	0.50 (0.34–0.74)***
Quintile 4	0.79 (0.70–0.93)**	0.42 (0.29–0.62)***
Quintile 5 (greenest)	0.71 (0.60–0.84)***	0.42 (0.29–0.61)***
Model 5: Adjusted for ethnicity, demographics, SES and health behaviours		
Quintile 2	0.94 (0.79–1.12)	0.64 (0.41–0.99)*
Quintile 3	0.80 (0.66–0.96)	0.72 (0.47–1.11)
Quintile 4	0.87 (0.72–1.05)	0.56 (0.37–0.86)**
Quintile 5 (greenest)	0.86 (0.71–1.06)	0.63 (0.41–0.97)*

†n=4436 maximum of 5 GSCEs.

‡n=3111 a-level or higher; physical activity comparison.

§n=6066 inactive.

¶n=1481 active.

*p<0.05; **p<0.01; ***p<0.001′

### Does physical activity mediateor moderate the relationship between green space and depression?

We first explored whether physical activity moderated relationships between green space and depression. We found a significant interaction within the 300m buffer zone (likelihood ratio test: p=0.04) only. In the unadjusted models, those in greener areas (quintiles 3–5) were significantly less likely to report depressive symptoms than those in the least green quintile for both groups (table 4). However, associations were stronger for active (OR 0.42, 95% CI 0.29 to 0.61) compared with inactive individuals (OR 0.71, 95% CI 0.60 to 0.84). While the pattern of findings persisted in the fully adjusted models, associations remained significant for the active individuals sample only. There was no interaction between physical activity and access to green space (results not shown).

We then explored the extent to which physical activity might mediate relationships between green space and depressive symptoms using binary mediation. For all three green space buffer zones, the indirect effect of green space on depression via physical activity was small, but significant (b=−0.01 for all, p=0.015, 0.025 and 0.030 for within 100, 300 and 500 m buffer zones, respectively). The direct effects were much larger (within 100 m buffer zone: b=−0.07, 95% CI −0.10 to −0.04; within 300 m: b=−0.09, 95% CI −0.11 to −0.06; within 500 m: b=−0.10, 95% CI −0.12 to −0.07, all p<0.001). The proportion of the total effect of green space on depressive symptoms accounted for by physical activity ranged from 5.6% (within 500 m buffer) to 7.8% (within 100 m buffer). The results suggest physical activity is a small, but significant, partial mediator of the effect of green space on depressive symptoms in pregnant women.

## Discussion

Our study is the first to explore the association between green space and depressive symptoms in pregnant women. We found a clear negative relationship between residential surrounding green space and likelihood of reporting depressive symptoms, with stronger associations seen in lower socioeconomic groups. The beneficial association with green space was also strongest for those who were physically active. Physical activity was not an important mediator of the relationship between green space and depressive symptoms.

In our sample, living in the greenest urban areas was associated with a reduction in the reporting of depressive symptoms, of nearly 18–23%. Our findings add to the limited evidence exploring the beneficial impact of green space during pregnancy and for the first time, show an impact on mental well-being. Calls for a ‘lifestyle medicine’ approach to prevent depression advocate the importance of both individual health behaviour change, and the environment.^8^ However, motivating individuals to change their behaviour can be difficult, and typically more affluent populations respond better to health promotion interventions than less affluent populations.^32^ Our findings suggest that the level of green space within a neighbourhood can have beneficial effects on mental well-being, independent of health behaviours, and therefore can benefit populations without requiring active behavioural change. Increasing green space should be prioritised by urban planners and policymakers, in addition to making continued efforts to promote healthy lifestyles.

Of interest is that the magnitude of the association between green space and depressive symptoms differed across quintiles, particular within smaller buffer zones. For example, within 100 m buffer zone, those in the greenest quintile were 18% less like to report depressive symptoms, compared with those within quintiles 3 and 4 who were 23% less likely to report depressive symptoms. It may be that there is a threshold effect for the beneficial effects of green space, beyond which point there are no more incremental benefits. This should be explored in future research.

Similar to other studies,^10^ ^11^ we found that beneficial effects of green space were stronger for women with lower SES defined by education, but not other SES indicators. It has been posited that disadvantaged groups may benefit more from green space interventions as they spend more time near their homes, resulting in more frequent use and interaction with the immediate neighbourhood environment.^11^ Therefore, from a health inequalities and environmental justice perspective, the above call to consider green space in healthy urban planning accords with the WHO's recognition that creating communities and neighbourhoods ‘that are designed to promote good physical and psychological well-being and that are protective of the natural environment are essential for health equity’.^33^

Few studies have explored whether the health impact of green space varies with ethnicity, although this has been hypothesised to be an important moderator.^34^ Unlike others,^11^ we did not find differences between ethnic groups. Further research should aim to explore possible ethnic differences in more detail; in particular, taking in to account green space use and quality issues.^35^

Finally, we explored the extent to which physical activity acted as mediator or moderator of the relationship between green space and depressive symptoms. Physical activity was a partial mediator, but the indirect effect was small. This is consistent with other recent studies,^16–18^ ^36^ ^37^ and suggests focusing attention on other mediators such as air quality, social contacts and stress reduction.^15^ Similar to Astell-Burt *et al,*^19^ we found that the benefits of green space on reporting of depressive symptoms were disproportionately greater for active individuals. There is review-level evidence that natural environment increases the positive effects of physical activity on well-being,^20^ so it is important to understand how to increase occurrence of physical activity in nature. A recent systematic review found only 12 studies which explored the impact of interventions to promote physical activity in urban green space.^38^ These authors reported ‘promising’ evidence that environmental improvements combined with physical activity promotion campaigns can be successful (compared with either environmental interventions or physical activity programmes alone). However, they also recognise the substantial methodological and theoretical limitations of the evidence, including poor descriptions of intervention content.^38^ There is a clear need for a systematic approach to the development and evaluation of interventions to promote utilisation and understanding of how to increase physical activity in these environments.

The main strengths of the current study were the large sample of ethnically diverse pregnant women from deprived areas, and comprehensive data that allowed us to control for a range of possible confounders of the relationship under investigation (including demographics, individual and neighbourhood SES indicators, and health behaviours).^27^ We were able to formally test whether physical activity mediated the relationship between green space and depressive symptoms in pregnant women. Finally we used an objective indicator of green space (amount and access), increasing comparability with other studies.

Limitations are recognised. First, data were cross-sectional, precluding causal inferences. This notwithstanding, the median years of residence at each household address prior to assessment was 3 (interquartile range 1–6), meaning that most women had several years of exposure to their neighbourhood prior to data collection. Second, by using a single NDVI map derived as the maximum greenness from two fixed time points, we assumed the spatial distribution of NDVI across our study region remained constant over the study period; however, our previous studies support the stability of the NDVI spatial contrast over seasons and years.^11^ ^12^ Third, our measure of green space did not take into account use and quality of green space, and our access to green space measure used Euclidean distance, rather than network distance to major green spaces. Fourth, the relatively high levels of deprivation and ethnic make-up of our sample may reduce generalisability to areas with greater affluence or less cultural diversity. Finally, our measure of depressive symptoms in pregnant women was constructed from a subset of items within the GHQ that are shown to relate the same construct across our different ethnic groups.^25^ Although such self-report may seem less preferable than more objective indicators such as health service records,^39^ we have previously found that the latter can underestimate prevalence of distress by almost half.^40^

Our finding that green space is associated with reduced depressive symptoms during pregnancy has a number of important implications for practice. Priority should be given to increasing urban green space within deprived communities, which may help to reduce health inequalities.^21^ Alongside these improvements, efforts should focus on encouraging active utilisation, particularly outdoor physical activity, to provide additional benefits. These changes will necessitate a coordinated approach to implementation which includes understanding of how to change behaviour at both a policy and individual level. Finally, future research should prioritise exploring how factors such as utilisation, quality and characteristics of green space impact on relationships with a view to refining understanding of the key mechanisms involved.

**What is already known on this subject**

- Depression during pregnancy can have negative health impacts on both mother and child. Lifestyle factors such as healthy eating, being physically active and avoiding illegal drugs can reduce the risk of depression. Living in greener areas might also ameliorate symptoms of depression. No previous research has shown a beneficial relationship between green space and depressive symptoms in pregnant women, a group at elevated risk of depression.

**What this study adds**

- We found that pregnant women living in greener environments were around 20% less likely to report depressive symptoms. The beneficial effect of green space was stronger in lower socioeconomic groups, and for those who were already physically active. There was little evidence that physical activity was the mechanism by which green space positively impacted on depressive symptoms. Efforts should be made to increase availability of green space at a policy level and utilisation of green space at an individual level.

## Supplementary Material

Web tables
