# Supplementary material for: The association between green space and depressive symptoms in pregnant women: moderating roles of socioeconomic status and physical activity
Source: J Epidemiol Community Health. 2015 Nov 11;70(3):253–9. doi: 10.1136/jech-2015-205954 (PMC4789818; doi:10.1136/jech-2015-205954)
Supplement: Web tables [file jech-2015-205954-s1.pdf]

Supplemental Table S1: Comparison of NDVI demographic variables with BiB full sample

	Current sample N=7547	Full baseline sample N=11396
NDVI		
100m	0.45 (0.11)	0.44 (0.11)
300m	0.48 (0.10)	0.47 (0.11)
500m	0.49 (0.10)	0.49 (0.10)
Missing	0	1341
Access to green space within 300m?		
No	1451 (19.2)	2382 (17.5)
Yes	6092 (80.7)	9867 (72.6)
Missing	0.1 (4)	1346 (9.9)
Ethnicity		
White British	3079 (40.8)	4487 (39.4)
Pakistani – English	2145 (28.4)	3204 (28.1)
Pakistani – Urdu/Mirpuri	1262 (16.7)	1994 (17.5)
Other	1061 (14.1)	1651 (14.5)
Missing		60 (0.5)
Maternal age		
<21 years	862 (11.4)	1298 (11.4)
≥21 & <34 years	5782 (76.6)	8740 (76.7)
≥35 years	903 (12.0)	1358 (11.9)
Missing	0	0
Marital status		
Married and living with partner	4904 (65.0)	7477 (65.6)
Not married and living with partner	1376 (18.2)	2023 (17.8)
Not living with partner	1267 (16.8)	1871 (16.4)
Missing	0	25 (0.2)
Education		
Low	4436 (58.8)	6810 (59.8)
High	3111 (41.2)	4556 (40.0)
Missing	0	30 (0.3)
Subjective poverty		
Struggling financially	2370 (31.4)	3634 (31.9)
Not struggling financially	5177 (68.6)	7728 (67.8)
Missing	0	34 (0.3)
Index of multiple deprivation		
Bottom national quintile	5973 (65.9)	7567 (66.4)
Missing	0	30 (0.3)
Drank alcohol during pregnancy or 3 months before		
Yes	2380 (31.5)	3481 (30.6)
No	5167 (68.5)	7882 (69.4)
Missing	0	3481 (30.6)

	Current sample N=7547	Full baseline sample N=11396
Smoking during pregnancy		
Yes	1348 (17.9)	1871 (16.5)
No	6199 (82.1)	9504 (83.6)
Missing	0	33 (0.3)

Supplemental figure 1. Flow diagram of study participants

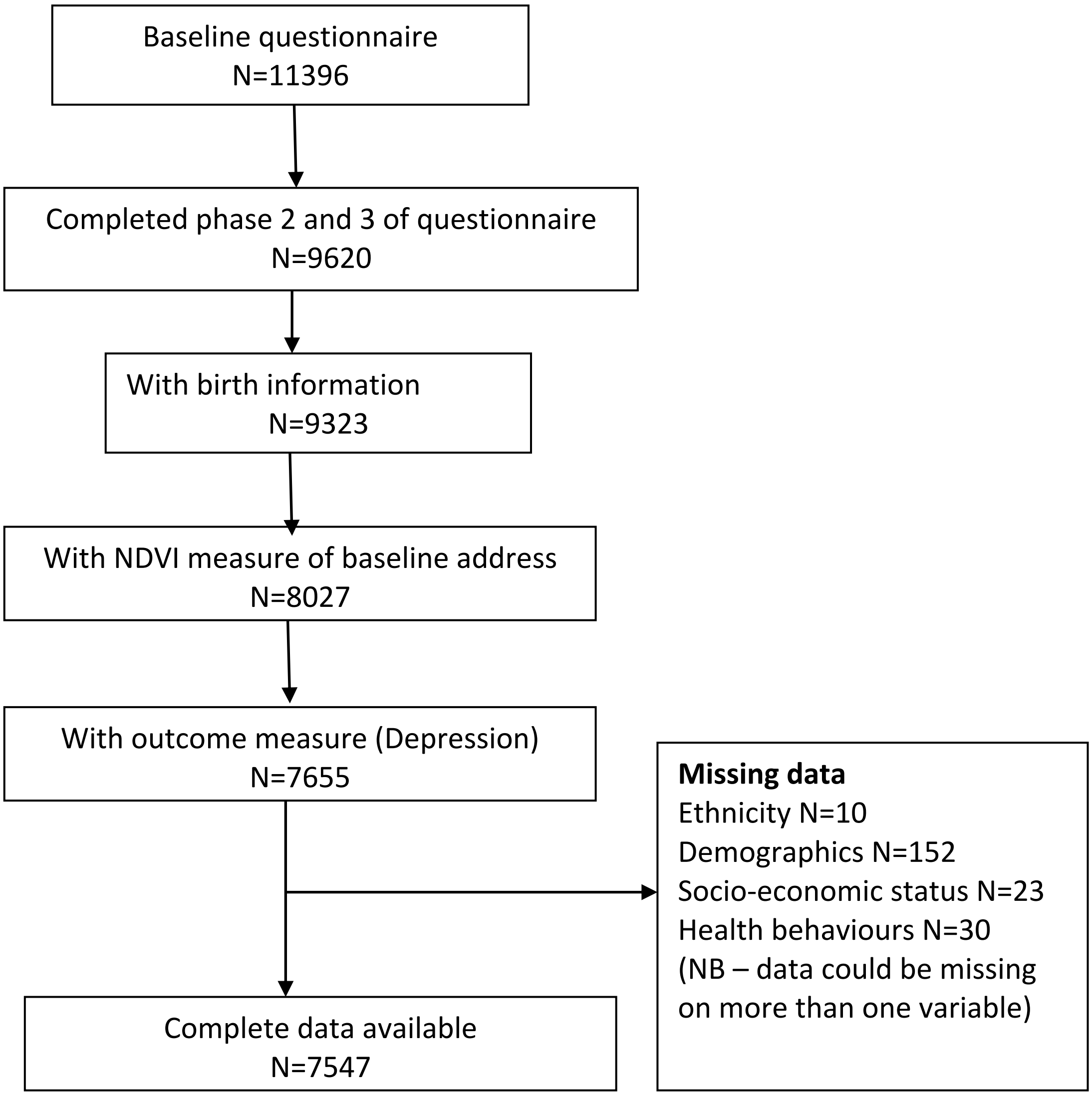

Supplemental Table S2. NDVI scores and distance to green space >0.5 hectares by reporting of depressive symptoms

	No depressive symptoms N=5017	Reporting depressive symptoms N=2530
NDVI 100m		
Quintile 1 (least green)	887 (60.8)	570 (39.1)
Quintile 2	946 (64.1)	530 (35.9)
Quintile 3	1059 (68.7)	482 (31.3)
Quintile 4	1065 (69.5)	467 (30.5)
Quintile 5 (most green)	1060 (68.8)	481 (31.2)
Mean (SD)	0.45 (0.11)	0.44 (0.11)
IQR	0.16	0.18
NDVI 300m		
Quintile 1 (least green)	886 (60.4)	580 (39.6)
Quintile 2	940 (63.6)	539 (36.4)
Quintile 3	1044 (69.3)	463 (30.7)
Quintile 4	1056 (68.4)	488 (31.6)
Quintile 5 (most green)	1091 (70.3)	460 (29.7)
Mean (SD)	0.48 (0.10)	0.47 (0.10)
IQR	0.16	0.17

NDVI 500m		
Quintile 1 (least green)	882 (60.3)	580 (39.7)
Quintile 2	934 (63.4)	543 (36.8)
Quintile 3	1047 (68.8)	475 (31.2)
Quintile 4	1034 (67.5)	498 (32.5)
Quintile 5 (most green)	1120 (72.1)	434 (27.9)
Mean (SD)	0.50 (0.10)	0.48 (.10)
IQR	0.16	0.17
Access to green space within 300m (NB 4 missing data)		
No	903 (62.2)	548 (37.8)
Yes	4110 (67.5)	1982 (32.5)
Distance (m) to green space	172 (130)	186 (134)
